# Mental illness attitudes and knowledge in non-specialist medical doctors working in state and private sectors

**DOI:** 10.4102/sajpsychiatry.v27i0.1592

**Published:** 2021-05-31

**Authors:** Yumna Minty, Mahomed Y.H. Moosa, Fatima Y. Jeenah

**Affiliations:** 1 Department of Psychiatry, Faculty of Health Sciences, University of the Witwatersrand, Johannesburg, South Africa

**Keywords:** mental illness, stigma, attitudes, mental health literacy, knowledge, doctors, healthcare workers, primary healthcare

## Abstract

**Background:**

An increasing number of South Africans utilise primary healthcare services (either in the state or private sector) for mental health concerns; hence, there is a need to objectively assess these doctors’ attitudes and knowledge of mental illness.

**Aim:**

To investigate aspects of knowledge and attitudes towards mental illness of a group of private and state-employed non-specialist medical doctors.

**Method:**

Doctors in the state sector who were working at a primary healthcare level and who were not working towards, or did not hold, a specialist qualification were considered eligible for the study. Doctors in the private sector who were working as general practitioners and who did not hold a specialist qualification were considered eligible for the study. Data were collected by means of a self-administered questionnaire. A link to the study questionnaire, information about the study, details of the researcher and matters pertaining to informed consent were emailed to potential participants.

**Results:**

Of the 140 practitioners who responded to the survey, 51.4% (*n* = 72) worked in the state sector, 41.4% (*n* = 58) worked in the private sector and 7.1% (*n* = 10) worked in both the state and private sectors (χ^2^_1_ = 45.31, *p* < 0.010). The majority (> 50%) of participants in all three groups had a positive attitude towards mental illness (χ^2^_2_ = 1.52, *p* = 0.468). Although there were no significant associations between attitude and socio-demographic characteristics (*p* > 0.05), male SS doctors reported feeling less comfortable when dealing with mentally ill patients (*p* = 0.015); SS doctors who did not have family contact with mental illness were less likely to feel that mentally ill patients did not pose a risk to others (*p* = 0.007), and PS doctors under the age of 35 years were more likely to feel adequately trained to treat mental illness (*p* = 0.026). The majority (> 50%) of participants in all three groups had an adequate level of knowledge of mental illness (modal scores = 10). There were no significant associations between knowledge and socio-demographic characteristics (*p* > 0.05).

**Conclusion:**

Despite the findings of a positive attitude and adequate knowledge of mental illness amongst the participants of this study, it is recommended that more targeted interventions are established to further improve mental health awareness and knowledge of doctors at both undergraduate and postgraduate levels of study.

## Introduction

The lifetime prevalence of developing a mental illness, as reported in the South African Stress and Health (SASH) study, is estimated to be about 30%.^1,2^ This is expected to become higher as the contributions from communicable diseases such as human immunodeficiency virus (HIV), non-communicable diseases,^3^ post-traumatic stress disorder and substance abuse^4^ become apparent.

Mental illness carries more stigma than any other illness, and sufferers are likely to be discriminated against more often and more significantly than the sufferers of other illnesses.^5^ Stigma combines a lack of accurate knowledge, prejudiced and negative attitudes and exclusionary or discriminatory behaviours to form a powerful driver for the social exclusion of persons with mental illness and the infringement of their rights and needs.^6^

Studies looking at mental health professionals’ perceptions of mental illness have found predominantly negative attitudes and the endorsement of restrictive and discriminatory practices towards the mentally ill.^7,8,9^ All of these studies used self-administered questionnaires as a means of evaluating mental illness perceptions, although the actual questionnaires and questions themselves differed amongst studies. A common perception of healthcare personnel, including medical doctors, is that people with mental illness pose a danger to others.^10,11,12,13^ Also expressed by healthcare workers is the belief that the mentally ill are blameworthy and are responsible for their conditions.^12,14,15^ Social distancing by society towards those with mental illness is common.^5,10,15^ It has also been found that certain mental illnesses such as schizophrenia attract more stigma than others.^7,16,17^

The extent to which patients may benefit from mental illness interventions is dependent not only upon available health resources but also on the level of knowledge and beliefs about mental illness within a community.^18^ Mental health literacy of healthcare workers is particularly important, as poor recognition or misdiagnosis of mental illness leads to delays in or no treatment, with subsequent poorer outcomes for the sufferers. Several studies have attempted to assess the mental health knowledge of healthcare workers and have found these to be poor or inadequate.^9,15,19,20,21^ Within South Africa, two studies have assessed nurses, in primary healthcare and psychiatrically trained nursing staff, and found inadequate levels of mental illness knowledge.^22,23^

Negative attitudes and stigma towards sufferers of mental illness form a barrier for treatment-seeking, for prevention of other diseases and conditions and for support in maintaining overall health.^24,25,26^

People with mental illness are at greater risk of premature death compared with those without mental illness^27^ because of poorer access to healthcare for associated physical illnesses^28^ and diagnostic overshadowing.^29^ Lack of accurate knowledge and the prevalence of misinformation contribute to stigmatising attitudes.

Therefore, poor knowledge of mental illnesses may contribute to not only poorer treatment but also perpetuation of stigma and hence reduced access to care, creating a vicious cycle of despair for the mental illness sufferer.

There is a significant body of data confirming that primary healthcare and generalist doctors, amongst other health professionals, hold negative attitudes regarding mental illness. Research also confirms that doctors have difficulty in diagnosing and instituting adequate treatment for mental illnesses. These factors lead to poorer healthcare for those with mental illness. Data on attitudes towards and knowledge of mental illness in South African samples of doctors are lacking, particularly with respect to knowledge of mental illness, hence the need for this study.

## Aim and scope of the study

This study investigated the attitudes and knowledge of non-specialist doctors in the public and private healthcare sectors towards mental illness. The specific objectives were to describe the sociodemographic characteristics, attitudes towards mental illness and knowledge of mental illness of the study populations. A secondary objective was to determine, if any, the factors associated with differences in attitudes and knowledge in doctors working in state and private sectors.

## Method

The study was a cross-sectional study. Doctors in the state sector who were working at a primary healthcare level and who were not working towards, or did not hold, a specialist qualification were considered eligible for the study. Doctors in the private sector who were working as general practitioners and who did not hold a specialist qualification were considered eligible for the study. Participants were not limited to doctors practising in Johannesburg.

### Data collection

Data were collected by means of a self-administered questionnaire. The questionnaire consisted of three parts. The first part of the questionnaire included information on the following: age, gender, number of years practising medicine, area of medical practice (state or private sector), contact with a close family member with mental illness. The second section of the questionnaire consisted of 10 statements describing attitudes towards mental illness in general. This component was developed after looking at other similar studies, which used Likert-scale questionnaires to assess mental illness attitudes.^10,30^ Ten statements covering stigma concepts such as social distance, negative attitudes, stereotyped beliefs and diagnostic overshadowing were compiled and used in the questionnaire. Respondents were asked to grade their level of agreement with the statements using a 5-point Likert scale (strongly agree, agree, uncertain, disagree and strongly disagree). The third section of the questionnaire consisted of 10 questions designed to assess the general knowledge of mental illness. Each question had three possible answer options (correct answer, incorrect answer and an option if the participant was unsure of the answer). This component was developed after perusing published mental illness literacy scales that assessed the general knowledge of mental illness.^31,32,33^ – these scales were not used in their entirety, with the aim of maintaining the brevity of the study questionnaire, so that participants would not find it tedious to complete. The questions were designed to cover factual aspects of mental illness that generalist doctors would likely encounter frequently, such as depression and anxiety, substance use, dementia, personality disorders and treatment non-adherence.

A link to the study questionnaire (an online questionnaire using a freely available online survey platform), information about the study, details of the researcher and matters pertaining to informed consent were emailed to potential participants. Participants accessed the questionnaire through the electronic link and submitted their responses electronically. The questionnaire was disseminated to potential participants as follows: (1) by the administrators of professional practitioner bodies including Independent Practitioners Association, the Islamic Medical Association, the General Practitioners Management Group and others; (2) by clinical managers at state facilities (primary healthcare clinics and district hospitals); and (3) by the researcher directly inviting eligible individuals to participate in the study or to extend the invitation to other colleagues. Data collection took place from December 2017 to September 2018. Data responses were originally recorded automatically in an online database and were then manually exported and recorded in a Microsoft Excel spreadsheet.

## Data analysis

Statistical analyses were conducted using Statistica (version 7; www.statsoft.com). Tests with two-tailed probability values and statistical significance were accepted when α ≤ 0.05. Data were described by means of proportions and percentages and illustrated using tables. Characteristics of the study populations were analysed and compared using chi-square contingency tests for all variables except age distribution. Age distribution was analysed using a generalised linear model (GLZ) with a Poisson error structure because the data set was large.

For the analysis of attitude and knowledge components, responses to statements and questions were scored to obtain numerical values for comparison. The overall attitude of the respondents towards mental illness was determined by allocating numerical values to the responses as follows: strongly agree = 2, agree = 1, uncertain = 0, disagree = -1, strongly disagree = -2. Certain statements were scored in reverse to reflect accurate portrayal of positive and negative attitudes. The modal score within each group was determined, as this was a representation of the attitude most commonly endorsed within that group. The scoring for knowledge questions was as follows: a correct response had a score of one and an incorrect response had a score of -1. A response of unsure had a score of zero. A higher score indicated a better knowledge of mental illness. Modal scores for answers to all of the questions were obtained. The sum of modal scores for each study group was compared using chi-square tests to determine any statistically significant difference in the three study groups.

Individual responses to each question were then compared using a GLZ model to determine if there were any such significant differences between the study groups. A GLZ model was used to determine if any of the recorded sociodemographic variables influenced differences in responses between the study groups. Wald *χ*^2^ outputs were reported for all GLZ analyses and estimates, and s.e. was reported for significant responses.

A GLZ model was used to determine if any of the recorded sociodemographic variables (age, gender, years of practice and having a family member with mental illness) influenced differences in responses between the study groups.

### Ethical considerations

Ethical approval was obtained from the University of the Witwatersrand Human Research Ethics Committee (protocol number M170733). Owing to the nature of the data collection, it was understood that written consent could not be obtained from each participant. An information leaflet with relevant information about consent for study participation was distributed along with the questionnaire, which made participants aware that tacit consent to participate was implied once an individual accessed the questionnaire and began the survey. Participants were informed that they had the option of not continuing with the questionnaire at any point or choosing not to submit their answers. In addition, they were not obliged to answer any question that they did not wish to.

## Results

Of the 140 practitioners who responded to the survey, 51.4% (*n* = 72) worked in the state sector, 41.4% (*n* = 58) worked in the private sector and 7.1% (*n* = 10) worked in the combined state and private sector (*χ*^2^_1_ = 45.31, *p* < 0.010).

### Sociodemographic characteristics

The mean age of the SS group was 34.4 years (range: 23–56 years), which was significantly lower than that of the PS group (44.6 years; range: 29–71 years) and the SPS group (44.3 years; range: 31–64 years) (Wald *χ*^2^ = 90.73, *p* < 0.010). The majority of participants in the SS group were in the < 35 year age group (52.3%, *n* = 38), between 35 and 45 years in the PS group (36.2%, *n* = 21) and between 35 and 45 years in the SPS group (50%, *n* = 5) (Table 1). Of note was that 17.2% (*n* = 10) of participants in the PS group were over 55 years of age compared with only 1.4% (*n* = 1) in the SS group.

**TABLE 1:** Frequency distribution of sociodemographic variables of the study population.

Sociodemographic variable	State sector (*n* = 72)		Private sector (*n* = 58)		State and private sector (*n* = 10)	
	*n*	%	*n*	%	*n*	%
**Age**						
< 35 years	38	52.3	8	13.8	1	10
35–45 years	21	29.2	21	36.2	5	50
46–55 years	11	15.3	13	22.4	1	10
> 55 years	1	1.4	10	17.2	2	20
**Gender**						
Male	24	33.3	35	60.3	8	80
Female	48	66.6	23	39.7	2	20
**Years of practice**						
< 5 years	28	40.3	0	0.0	0	0
5–10 years	18	25.0	10	17.2	2	20
11–15 years	16	22.2	17	29.3	5	50
16–20 years	5	6.9	10	17.2	1	10
> 20 years	5	6.9	20	34.5	2	20
**Family member with mental illness**						
Yes	30	41.7	27	46.6	4	40
No	35	48.6	24	41.4	5	50
Unsure	12	16.7	5	8.6	1	10

Majority of the participants were male in the PS group (60.3%, *n* = 35) (*χ*^2^ = 90.73, *p* = 0.115) and the SPS group (80%, *n* = 8) (*χ*^2^ = 3.60, *p* = 0.060), whilst the majority were female in the SS group (66.6%, *n* = 48) (*χ*^2^_2_ = 8.00, *p* = 0.005) (Table 1). The majority of participants in the SS group were practising for less than 5 years (40.3%, *n* = 28), for more than 20 years in the PS group (34.5%, *n* = 20) and for between 11 and 15 years in the SPS group (50%, *n* = 5) (*χ*^2^ = 12.96, *p* = 0.044). Significantly more respondents in the SS group were employed for less than 5 years and significantly fewer for 20 years or more, compared with the other two groups (*χ*^2^ = 27.71, *p* < 0.001) (Table 1). There were no statistically significant differences with respect to personal or family contact with mental illness (*p* > 0.05).

### Attitudes towards mental illness

The majority of the participants (> 50%) in all three groups agreed with the following statements in the questionnaire: ‘people with mental illness rarely pose a risk to their families and communities’, ‘employers should hire a person with a managed mental illness if he or she is the best person for the job’, ‘I feel that I am adequately trained and have sufficient knowledge to be able to diagnose and initiate treatment in a person with mental illness’ and ‘I would not mind if a person with mental illness lived next door to me’ (Table 2).

**TABLE 2:** Frequency distribution of responses to the attitude statements in the study population.

Attitude statements	State sector (*n* = 72)		Private sector (*n* = 58)		State and private sector (*n* = 10)	
	*n*	%	*n*	%	*n*	%
**1. People with mental illness rarely pose a risk to their families and communities**						
Strongly agree	4	5.6	7	12.1	0	0
Agree	36	50.0	32	55.2	5	50
Uncertain	4	5.6	3	5.2	1	10
Disagree	23	31.9	8	13.8	4	40
Strongly disagree	5	6.9	7	12.1	0	0
**2. I am more comfortable treating someone with a physical illness than a mental illness**						
Strongly agree	5	6.9	3	5.2	1	10
Agree	47	65.3	23	39.7	5	50
Uncertain	2	2.8	3	5.2	0	0
Disagree	15	20.8	24	41.4	4	40
Strongly disagree	3	4.2	5	8.6	0	0
**3. Employers should hire a person with a managed mental illness if he/she is the best person for the job**						
Strongly agree	20	27.8	26	44.8	2	20
Agree	46	63.9	29	50.0	5	50
Uncertain	4	5.6	0	0.0	1	10
Disagree	2	2.8	2	3.4	0	0
Strongly disagree	0	0.0	1	1.7	2	20
**4. Healthcare providers have a role in assisting mentally ill patients in becoming active members of their communities**						
Strongly agree	33	45.8	32	55.2	4	40
Agree	37	51.4	24	41.4	5	50
Uncertain	2	2.8	1	1.7	1	10
Disagree	0	0.0	0	0.0	0	0
Strongly disagree	0	0.0	0	0.0	0	0
**5. Most people with mental illness don’t try hard enough to get better**						
Strongly agree	0	0.0	1	1.7	0	0
Agree	13	18.1	14	24.1	0	0
Uncertain	7	9.7	4	6.9	0	0
Disagree	45	62.5	35	60.3	8	80
Strongly disagree	7	9.7	4	6.9	2	20
**6. I sometimes struggle to feel compassion for a person with mental illness**						
Strongly agree	19	26.4	0	0.0	0	0
Agree	0	0.0	16	27.6	4	40
Uncertain	4	5.6	4	6.9	0	0
Disagree	41	56.9	25	43.1	2	20
Strongly disagree	8	11.1	13	22.4	4	40
**7. If a person with mental illness complained of physical symptoms (e.g. headache, nausea), I would likely attribute this to their mental illness.**						
Strongly agree	1	1.4	0	0.0	0	0
Agree	18	25.0	6	10.3	2	20
Uncertain	6	8.3	0	0.0	0	0
Disagree	42	58.3	43	74.1	6	60
Strongly disagree	5	6.9	8	13.8	2	20
**8. I feel that I am adequately trained and have sufficient knowledge to be able to diagnose and initiate treatment in a person with mental illness.**						
Strongly agree	4	5.6	7	12.1	0	0
Agree	29	40.3	29	50.0	6	60
Uncertain	11	15.3	4	6.9	1	10
Disagree	26	36.1	17	29.3	3	30
Strongly disagree	2	2.8	1	1.7	0	0
**9. I would not mind if a person with mental illness lived next door to me.**						
Strongly agree	4	5.6	9	15.5	3	30
Agree	58	80.6	43	74.1	6	60
Uncertain	5	6.9	2	3.4	0	0
Disagree	5	6.9	4	6.9	1	10
Strongly disagree	0	0.0	0	0.0	0	0
**10. I sometimes perceive people with mental illness as being weak or lacking in willpower and self-discipline.**						
Strongly agree	0	0.0	1	1.7	0	0
Agree	17	23.6	8	13.8	1	10
Uncertain	3	4.2	3	5.2	1	10
Disagree	41	56.9	38	65.5	5	50
Strongly disagree	11	15.3	8	13.8	3	30

The majority of participants (> 50%) in all three groups disagreed with the following statements posed in the questionnaire: ‘most people with mental illness don’t try hard enough to get better’, ‘I sometimes perceive people with mental illness as being weak or lacking in willpower and self-discipline’ and ‘if a person with mental illness complained of physical symptoms, I would likely attribute this to their mental illness’ (Table 2).

The only significant difference found in responses for individual questions was that participants in the SS group were more likely to agree that they were more comfortable treating someone with a physical illness than a mental illness, when compared with the PS group (Wald *χ*^2^_1_ = 15.691, *p* = 0.047).

The most overall positive attitude towards mental illness was observed in the PS group (total modal score = 11) followed by the SS group (total modal score = 8) and the SPS group (total modal score = 6). The differences between the three groups were not statistically significant (*χ*^2^_2_ = 1.52, *p* = 0.468).

### Knowledge of mental illness

The majority of participants in all three groups (> 50%) gave the following correct responses to questions in the survey: ‘psychotherapy may be the most beneficial intervention in personality disorders’, ‘someone who has a close family member with depression is at risk of developing depression’, ‘people with schizophrenia who do not take their treatment regularly most likely do so because they do not recognise that they have an illness’, ‘people with post-traumatic stress disorder are at high risk for developing alcohol abuse’, ‘a woman with borderline personality disorder who frequently becomes suicidal because of an ongoing fear is most likely fearful of abandonment’ and ‘an elderly patient who is brought to the doctor by her family as she has become difficult and unmanageable at home over the past few months, hiding things away and accusing family of stealing things from her, is most likely suffering from dementia’ (Table 3).

**TABLE 3:** The frequency distribution of responses for each of the questions in the study population.

Knowledge statements	State sector group (*n* = 72)		Private sector group (*n* = 58)		State and private sector (*n* = 10)	
	*n*	%	*n*	%	*n*	%
**1. People who have had an episode of depression are most at risk for a psychotic disorder or another episode of depression**						
Correct	68	94.4	58	100.0	10	100
Incorrect	4	5.6	0	0.0	0	0
Unsure	0	0.0	0	0.0	0	0
**2. Psychotherapy may be the most beneficial intervention in personality disorders or learning disorders**						
Correct	58	80.6	50	86.2	9	90
Incorrect	11	15.3	7	12.1	1	10
Unsure	3	4.1	1	1.7	0	0
**3. A man, at the airport, who is talking and laughing very loudly, passing out money to people and talking about his plans to fly around the world, is most likely suffering from mania or delirium**						
Correct	64	88.9	51	87.9	10	100
Incorrect	8	11.1	6	10.3	0	0
Unsure	0	0.0	1	1.7	0	0
**4. Someone who has a close family member with depression is at risk of developing depression or dementia**						
Correct	57	79.2	47	81	7	70
Incorrect	14	19.4	9	15.5	2	20
Unsure	0	0.0	2	3.4	1	10
**5. People with schizophrenia who do not take their treatment regularly, most likely do so because they do not recognise that they have an illness or they believe that medication is ineffective**						
Correct	49	68.1	45	77.6	7	70
Incorrect	21	29.2	12	20.7	3	30
Unsure	2	2.7	1	1.7	0	0
**6. People with post-traumatic stress disorder are at high risk of developing dementia or alcohol abuse**						
Correct	57	79.2	44	75.9	8	80
Incorrect	13	18.1	13	22.4	2	20
Unsure	2	2.7	1	1.7	0	0
**7. A woman with borderline personality disorder who frequently becomes suicidal because of an ongoing fear is most likely fearful of abandonment or gaining weight**						
Correct	70	97.2	56	96.6	8	80
Incorrect	2	2.8	0	0.0	2	20
Unsure	0	0.0	2	3.4	0	0
**8. A woman who has been isolating herself from friends and family and no longer pursues hobbies and interests as before is most likely suffering from generalised anxiety disorder or depression**						
Correct	65	90.3	49	84.5	10	100
Incorrect	7	9.7	9	15.5	0	0
Unsure	0	0.0	0	0.0	0	0
**9. Mental illness can most often be attributed to chemical imbalances or structural damage to the brain or poor self-control**						
Correct	70	97.2	55	94.8	10	100
Incorrect	2	2.8	3	5.2	0	0
Unsure	0	0.0	0	0.0	0	0
**10. An elderly patient is brought to the doctor by her family as she has become difficult and unmanageable at home over the past few months, hiding things away and accusing family of stealing things from her. She is most likely suffering from dementia or delusional disorder**						
Correct	59	81.9	50	86.2	8	80
Incorrect	12	16.7	7	12.1	1	10
Unsure	0	0.0	0	0.0	1	10

The majority of the doctors in the SS group (> 50%) and all of the doctors in the PS group and SPS group responded correctly to the question, which stated that people who have had one episode of depression are most at risk for another episode of depression.

In addition, the majority of doctors in the SS and PS groups (> 50%) gave correct responses to the following questions: ‘a man at the airport who is talking and laughing very loudly, passing money to people and talking about his plans to fly around the world is most likely suffering from mania’, ‘a woman who has been isolating herself from friends and family and no longer pursues hobbies and interests as before is most likely suffering from depression’ and ‘mental illness can most often be attributed to chemical imbalances or structural damage to the brain’.

The sum of the modal scores for all three groups was the same, indicating that there was no statistical difference between the knowledge scores of the three groups.

### Association between sociodemographic characteristics and attitude and knowledge of mental illness

The SPS group was excluded from this analysis because of the small sample size. There was no statistically significant difference in the overall attitude towards mental illness between the SS and PS groups with respect to age (Wald *χ*^2^_1_ = 9.564, *p* = 0.089), gender (Wald *χ*^2^_1_ = 0.636, *p* = 0.425), duration of practice (Wald *χ*^2^_1_ = 0.292, *p* = 0.999) and having a family member with mental illness (Wald *χ*^2^_1_ = 3.248, *p* = 0.197).

There were also no significant associations between overall knowledge score between the SS and PS groups and any of the sociodemographic variables (*p* > 0.05), as well as no significant influences of sociodemographic variables on differences in individual responses to any of the knowledge questions (*p* > 0.05).

## Discussion

### Attitudes towards mental illness

The study found that the overall attitude towards mental illness of both SS and PS doctors was positive. This is similar to the findings of published international studies.^34,35,36,37^

In one such study, Wang et al.^38^ found that over 80% of study respondents (who were Chinese non-psychiatric hospital doctors) felt that they had a responsibility towards their patients in managing their psychological distress. To the best knowledge of the researchers, there are no published studies amongst non-specialist medical doctors working in South Africa. However, Eksteen et al.^39^ found that psychiatrists had the least stigmatising attitudes when compared with pre-clinical and post-clinical medical students. Mausling et al.^40^ showed that second-year medical students had positive attitudes towards psychiatric illness and psychiatry, whilst Dube et al.^23^ demonstrated that nurses working in primary healthcare clinics were positive about the management of psychiatric patients. Studies have also shown positive attitudes in other healthcare workers, such as medical and nursing students^41^ and nurses, social workers and healthcare assistants.^42^ Similar to this study, it appears from the literature that doctors and other healthcare professionals exhibit positive attitudes towards people with mental illness.

However, there is also a significant body of literature, which shows that doctors have negative attitudes towards mental illness.^11,13,43,44^

One such review,^36^ looking at stigma and discrimination towards mental illness in healthcare professionals found negative attitudes in psychiatrists, nurses, psychologists and medical students; it appeared that the most negative attitudes were described in psychologists. It was also found that chronically ill patients with more hospital admissions were viewed more negatively than patients with milder symptoms. In a South African study, Jury^45^ described negative attitudes towards mental illness in specialist doctors practising in state sector teaching hospitals. In addition, a study of South African nurses by Mavundla et al.^46^ found that participants held mainly negative attitudes towards the mentally ill.

The positive attitudes found in this study could have a number of explanations. It may have been partly as a consequence of improved training and exposure to psychiatry at the undergraduate level, and exposure to psychiatric patients during the internship period.^47^ In addition, there is increasing recognition that the contribution of mental illness to the global disease burden is on the rise^48^ with a consequence of this being that healthcare workers have increased exposure to mental illness and thus may have more positive perceptions. With mental health issues such as the Life Esidimeni tragedy and the recent suicides of prominent figures being widely covered in the mainstream and social media, awareness and advocacy for mental health has improved. This has likely led to a more sympathetic stance towards mental illness amongst doctors and in other sectors of the general population. Mental illness is also on the rise within the medical profession, with recent statistics showing a considerable prevalence in doctors (at the time of writing this article, statistics for mental illness in South African doctors were not available).^49,50^

This may be another contributing factor to positive attitudes, as doctors may be more likely to have a favourable opinion of mental illness if they have personally experienced such symptoms.

Although the difference was not statistically significant, this study found that SS doctors were slightly less positive towards mental illness than their PS counterparts. Similar findings have been reported in other studies.^7,9,51^ Jury’s^45^ study of specialist doctors practising in South African state sector teaching hospitals described negative attitudes towards mental illness, in particular towards schizophrenia and borderline personality disorder. There are, however, published studies with contrasting findings.^15,52,53^

The less positive attitude amongst SS doctors may be because of inadequate exposure to mental illness because of a lack of integration of mental healthcare at primary healthcare level, a demanding workload and a tendency to focus consultations on physical complaints with less time devoted to mental health complaints. In addition, as the SS doctors in this study were generally younger and less experienced than the PS doctors, it is likely that age and lesser experience contributed towards less positive attitudes, as in other studies.^11,54^ Studies have shown that increased exposure to mentally ill people leads to more positive attitudes.^55,56,57^ It is also likely that PS doctors are more open to advocating for their mentally ill patients because of the lesser demands and time constraints of their work as compared with state-employed doctors. Within private practice, it is easier to develop long-lasting and enduring doctor–patient relationships, as patients most often consult with the same doctor multiple times. This is unlike the state sector, where many doctors work in a single department and doctors rotate through different units, hospitals or clinics, meaning that they often do not consult regularly with the same patient. This fragmented relationship may exacerbate feelings of social distance and contribute to less positive attitudes towards patients with mental illness.

### Sociodemographic variables associated with attitudes towards mental illness

This study found that the factors of age, gender, duration of practice and having a family member with mental illness were not significantly associated with overall attitudes towards mental illness. Similarly, Sujaritha et al.^52^ and Sri et al.^53^ reported that gender and level of qualification did not influence mental illness attitudes, whilst Vistorte et al.^58^ found that gender, age, training and years of experience did not hold any association with stigmatising attitudes.

This is contrary to the findings of other published studies, which have reported both positive and negative associations with demographic characteristics. Female doctors have been found to be more positive and much less stigmatising than male doctors.^45,59,60,61^ Stuber et al.^62^ noticed that younger female mental health professionals were less stigmatising than older male professionals. Ewalds-Kvist et al.^61^ reported that even though older individuals were more open-minded towards mentally ill people, they were less likely to be supportive of community psychiatric services and integration. Those with more clinical experience^63^ or a family member or friend with mental illness^7,11^ were more sympathetic towards the mentally ill. However, Chandramouleeswaran et al.^55^ found that personal contact with an individual with a psychiatric condition did not increase the positivity of mental illness attitudes.

Within available literature, the overall impression is that there is no consistent sociodemographic predictor of mental illness attitudes as the relationship between variables and attitudes differs across studies, suggesting that these interactions may be complex and multifaceted. The interplay between demographic factors and cultural influences is also likely to play a role in how mentally ill people are viewed by others.

### Knowledge of mental illness

The study found that doctors had adequate knowledge of mental illness, as evidenced by the high modal scores. Similar findings have been reported in other studies.^64,65,66^

In contrast, some studies have reported low levels of mental illness knowledge amongst doctors,^15,19,67,68^ particularly in those who had little or no formal psychiatric training. It would appear that non-psychiatric doctors are, at the very least, comfortable in diagnosing common mental illnesses such as depression but feel less confident in being able to treat mentally ill patients effectively.^69,70^ Inadequate mental illness knowledge appears to be prevalent in medical students and nurses as well.^20,22^

The findings of adequate levels of knowledge in this study may have been, in part, because of training and exposure to psychiatry in medical teaching facilities in South Africa. South African universities have established training programmes for medical and psychiatric teaching, with four of South Africa’s medical schools being ranked in the top 300 best universities globally for clinical medicine.^71^ Another explanation is that the research questions may have been too simple and straightforward and focused only on diagnosis and symptomatology – this is a retrospective finding. As literature shows, non-psychiatric doctors reportedly have most difficulty with instituting treatment for mental illness and appropriate pharmacotherapy. Hence, there appears to be significant gaps in primary care and non-specialist doctors’ knowledge of mental illness with regard to interventions. Including questions on treatment and pharmacotherapy may have yielded different, albeit more representative, results.

### Sociodemographic variables associated with knowledge of mental illness

Overall, no significant associations between sociodemographic variables and mental illness knowledge could be made in any of the study groups. Significant associations were made in other studies. Younger age,^66^ engagement in mental health training not long before participation in a research study,^69,70^ as well as being a family physician (compared to GPs or other specialists)^64^ are associated with better diagnostic and practical knowledge. Aruna et al.^20^ found that the level of knowledge in undergraduate medical students increased with progressive years of study, such that students in their final years had better mental health literacy than those in their first year of study. The general finding appears to be that doctors who engaged in some form of compulsory psychiatric training, over and above standard medical school teaching, have better knowledge of mental illness and are thus better able to manage psychiatric conditions.

It is noteworthy that positive attitudes towards mental illness appear to be associated with a better knowledge of mental illness and self-perceived competence in dealing with mental illness.^55,58^

Negative attitudes appear to be associated with a lack of mental health training and a self-perceived lack of competence in managing mental illness.^15^ Also, better knowledge appears to be associated with a more positive attitude. Whilst it may not be possible to discern whether better knowledge is a consequence of positive attitudes or vice versa, it seems clear that the two share a significant association and both are likely to have an important contribution towards patients receiving adequate mental healthcare. Therefore, it stands to reason that in order to reach an end goal of improved healthcare for mentally ill patients, both doctors’ attitudes and mental health knowledge need to be adequately addressed.

Focusing on improving both factors (attitudes and mental health knowledge) is likely to lead to significantly better health outcomes for the mentally ill. Training of non-specialist doctors, focused on recognition and management of mental illnesses, may contribute to reducing the stigma of mentally ill patients within healthcare services. Consequently, this may lead to an increased willingness for families and patients to seek help, leading to earlier diagnosis, better recognition of illnesses and improved outcomes. A focus on fostering positive attitudes towards mental illness in non-specialist and primary healthcare doctors may make doctors more receptive to learning more about mental illnesses. This, in turn, may mean that doctors are willing to spend more time with mentally ill patients to determine their psychiatric symptoms and stressors and manage their illnesses better.

## Limitations of the study

An important limitation of this study is selection bias. Participants in the study were selected through convenience sampling and doctors who already held positive attitudes towards mental illness may have been more likely to complete the survey compared with doctors who would have expressed negative attitudes, thereby skewing the study’s findings in favour of a more positive result. Another limitation is that the questionnaire used in the study was developed for the purposes of this study only and has not been validated or used in any other study – this may affect the comparison of the study’s findings to other research. As previously mentioned, the questions used to assess mental illness knowledge may have been too simplistic and easy, thereby affecting the results.

## Recommendations

Although positive findings were demonstrated overall, some doctors seem to have negative attitude towards the mentally ill. Other South African samples have displayed overall negative attitude towards mental illness, which lends weight to the necessity of anti-stigma interventions for South African healthcare professionals. A number of stigma-reduction interventions have been researched and evaluated; these involve approaches at varying levels, including interpersonal, institutional and structural levels.

Whilst the doctors who participated in this study appear to have a good knowledge of mental illness, improving on all aspects of psychiatric knowledge in non-specialist doctors has the potential to improve care at a primary healthcare and generalist level. Several educational programmes and teaching interventions to improve mental illness outcomes at the primary healthcare level have been developed and may be of benefit. These include the WHO’s Mental Health Gap Action Programme, individualised training, e-learning approaches, workshops and continuous professional development and mentoring programmes coordinated by specialists. Other interventions include increasing the length of time that medical students spend in psychiatric rotations, increasing exposure to stable patients and rehabilitative services for the mentally ill and introducing specialised and specific anti-stigma teaching into psychiatric rotations.^47,72^ Reducing mental illness stigma and improving mental health knowledge can be achieved simultaneously through improved and consistent education and training at an undergraduate level, as well as upskilling and continuous professional development for qualified healthcare professionals.

## Conclusion

This study showed that, in spite of limitations of application of the study tool, non-specialist doctors in both state and private sectors generally held positive attitudes towards mental illness and displayed adequate levels of knowledge, particularly with respect to the symptoms and diagnosis of mental illness. Doctors working in the private sector were slightly more positive in their attitudes than state-employed doctors.

Amongst the state-employed doctors, male doctors felt more comfortable with treating physical illnesses than mental illnesses, whilst those who were practising for more than 10 years and who had no close contact with mental illness had less positive attitudes.

To the best knowledge of the researcher, no other studies looking at attitudes and knowledge of mental illness in non-specialist doctors in South Africa have been performed. The findings of this study are important and relevant when one considers the high burden of mental illness in South Africa, as well as the prevalence of mental illness in patients consulting at the primary healthcare facilities, which are often the first port of call for seeking treatment and assistance.
